# Air Pollution and Household Medical Expenses: Evidence From China

**DOI:** 10.3389/fpubh.2021.798780

**Published:** 2022-02-07

**Authors:** Li Zhou, Qian Zhong, Jingjing Yang

**Affiliations:** School of Finance, Guangdong University of Foreign Studies, Guangzhou, China

**Keywords:** CFPS, air pollution, household medical expenses, household consumption, Qinling Mountains-Huaihe River Line

## Abstract

By matching air quality index (AQI) data with the household data from China Family Panel Studies (CFPS), we identify the impact of air pollution on household medical expenses from a micro perspective. The results show that higher air pollution will increase household medical expenses and change household consumption structure. This effect is still significant after controlling for cities' relevant household and individual characteristics and economic characteristics. Under different educational backgrounds, income, hukou, gender, and other conditions, air pollution will significantly reduce medical spending. For those females in the urban areas with higher education backgrounds and higher income, the spending elasticity of air pollution is more significant than other corresponding groups. And air pollution will promote medical expenses through stronger individuals' environmental awareness, poor health conditions, bad emotional status, and positive risk aversion. Furthermore, we find that the impact of air pollution on healthcare spending remains significant after instrumental variables regression and geographical regression based on the Qinling Mountains-Huaihe River Line.

## Introduction

With the rapid economic growth in developing countries, environmental degradation, especially air pollution, raises more public attention. Many researchers have confirmed the thread of air pollution on individual health (1, 2). In China, air pollution ranks 4th among the ten leading causes of death (3). Severe air pollution has produced heavy pressure on the household life and health (4). As a response, people's consumption structure and the proportion of medical expenses may be changed.

Some articles study the impact of environmental pollution on household consumption from the macro perspective. Grossman (5) proposes health capital and health production functions. Cropper (6), Gerking and Stanley (7) introduce environmental pollution variables into production functions and believe that pollution will affect the health depreciation rate, laying an essential foundation for the follow-up research. Narayan and Narayan (8) find that carbon monoxide, hydrogen sulfide, and other pollutants are positively correlated with medical and health expenses for OECD member countries. Currie and Walker (9) believe that electronic road toll systems lower the premature birth rate by reducing the concentration of ambient air pollutants, improving the health of newborns, and alleviating the economic burden.

More and more scholars use household microdata to explore how a household changes its consumption behavior under air pollution. Patankar and Trivedi (10) uncover that individuals, especially the poor, mainly bear related medical expenses caused by air pollution. Isen et al. (11) believe that those exposed to the polluted environment in childhood will affect the income level in adulthood through the healthy capital, thus influencing consumption in the life cycle. Pi et al. (4) focus on air quality influence on medical insurance expenditure using China Health and Retirement Longitudinal Study (CHARLS). They find that there is a negative correlation between air pollution and medical insurance expenditure. However, their dataset only includes middle-aged and elderly households and thus cannot fully explain this issue.

Although the above literature has pointed out a significant effect of air pollution on medical expenses and consumption structure, more heterogeneous effects and mechanisms remain further explained. Besides, the possible endogenous problems have not received enough attention in the above literature. Existing articles have confirmed the impact of household consumption on air pollution (12), reminding us of the potential endogeneity between air pollution and household consumption. Thus, an empirical framework for causal identification is needed to address possible endogenous problems. Under this background, how the increasingly severe air pollution impacts household medical and healthcare consumption and the heterogeneous effect among individuals are two crucial issues to be explored.

To answer the two above questions, by matching China's AQI data at the city level with household data, we discuss the causal effect of air pollution on household medical and health care expenditure from a micro perspective. We find that air pollution will significantly increase household medical and health care expenses. This effect is still significant after controlling for relevant household and individual characteristics and cities' economic variables. Under different educational backgrounds, income, hukou, gender, and other conditions, air pollution significantly affects consumer spending. For those females in the urban areas with higher education backgrounds and higher income, the spending elasticity of air pollution is more effective than other corresponding groups. Furthermore, we find that the impact of air pollution on household spending is particularly substantial in cities north of the Qinling Mountains-Huaihe River Line. Finally, we empirically illustrate how air pollution affects individual medical expenses through physical health, environmental awareness, risk attitude, and emotional status.

Our work makes three primary contributions. First, this paper uses a more complete data set and thoroughly discusses the heterogeneous impact of air pollution on medical costs. Different from Pi et al. (4), which only study middle-aged and older people, we focus on the householder between 17 and 95 years old, and we find that the conclusions obtained using the complete data set are very different from those of Pi et al. (4). Second, this paper empirically examines the mechanisms through which air pollution promotes medical expenses in a comprehensive framework from the household micro perspective, including stronger individuals' environmental awareness, poor health conditions, bad emotional status, and positive risk aversion. Third, this paper profoundly discusses possible endogenous issues. Through instrumental variable regressions, geographical regression design, we confirm the robustness of the preliminary empirical results, thus providing more empirical analysis methods for this research field.

The rest of this paper is organized as follows. Data Sources and Descriptive Statistics section describes the data and econometric models. Empirical Results section presents the empirical results, including the basic regressions, heterogeneity analysis, endogenous discussions, and possible channels. We discuss the policy implications and conclude in Conclusion section.

## Data Sources and Descriptive Statistics

### Data

The data utilized in this paper is the matched data from households and cities. Household-level data is from China Family Panel Studies (CFPS) in 2014 and 2016, and we apply the sample matching method to construct panel data with two periods. In this paper, the primary dependent variables are total household consumption and health care expenditure, of which the total consumption is the sum of eight items including food, clothing, daily necessities and services, transportation and communication, education and entertainment, housing, health care, and other supplies and services.

Among many types of pollution, air pollution is most intuitively felt. Thus it is most practicable to choose indicators that can measure air pollution. This article uses daily standardized AQI data published by the Ministry of Ecology and Environment of the People's Republic of China to reflect air pollution in the city where the individual currently lives. Considering household consumption is the annual data, we aggregate the daily AQI yearly to ensure consistent statistics when matching the data.

This paper uses the consumer price sub-index (divided by 100) in multiple provinces for the various commodities prices. The first seven commodities categories in each province are based on their respective price index. The missing price index for “other goods and services” is substituted by the consumer price index (CPI).

According to the Life-cycle hypothesis, household income, net assets, and debt are essential factors affecting household consumption, required to control their impact on consumption in the empirical test. The household income includes three major parts, wages and operating income, property income, and transfer income. Meanwhile, we also add the following control variables, individual characteristics (age of householder, gender, marital status, education background, risk attitude), household-level characteristic variables (whether they hold their own house, family size, child ratio, elderly ratio), and cities' economic variables.^1^ To eliminate the heteroscedasticity, extreme outliers, and inflation factors, we use the CPI index to adjust the variables based on 2014, including value variables such as household consumption, household income, household debt, and winsorizing at 1% and taking the logarithm. Descriptive statistics of the variables are shown in Table 1.

**Table 1:** Descriptive statistics.

**Variables**	**Obs**	**Mean**	**Std. Dev**	**Min**	**Max**
Lnpce	12,924	50,241	52,908	2,950	331,960
Lnmed	13,281	4,957	9,391	0	62,060
Lndaily	13,172	7,010	21,231	0	160,860
Lndress	13,148	2,345	3,035	0	20,000
Lneec	13,237	4,768	8,107	0	45,000
Lnfood	13,222	16,737	14,242	420	72,000
Lnhousing	13,156	7,325	17,253	0	125,680
Lntrco	13,104	4,209	5,034	0	28,800
Lnother	13,252	674	1,510	0	11,000
AQI	13,281	0.238	0.055	0.104	0.381
PM2.5	13,281	0.215	0.058	0.048	0.335
PM10	13,281	0.238	0.065	0.038	0.411
SO_2_	13,281	0.250	0.067	0.121	0.415
NO_2_	13,281	0.324	0.058	0.138	0.497
CO	13,281	0.277	0.074	0.079	0.512
O_3_	13,281	0.368	0.054	0.197	0.506
AQI100_num	13,281	52	33	0	144
AQI150_num	13,281	19	18	0	94
AQI200_num	13,281	8	10	0	62
Age	13,281	52	14	17	95
Eduy	13,281	8	4	0	22
Work_dum	13,281	0.726	0.446	0	1
Health_dum	13,281	0.821	0.384	0	1
Social_insurance	13,281	0.927	0.260	0	1
Urban	13,281	0.507	0.500	0	1
Marry_dum	13,281	0.867	0.339	0	1
Gender	13,281	0.508	0.500	0	1
Ownership	13,281	0.863	0.344	0	1
Risk_prefer	13,281	0.164	0.371	0	1
Child_ratio	13,281	0.124	0.165	0	1
Old_ratio	13,281	0.176	0.313	0	1
Familysize	13,281	4	2	1	17
Lnincome	13,281	51,624	47,715	800	240,500
Lntotal_asset	13,281	474,865	749,621	0	4,214,000
Lnhouse_debt	13,281	17,955	62,932	0	450,000
Lnnonhouse_debt	13,281	6,928	25,820	0	200,000
Green	13,281	39.610	8.061	3.070	92.870
GDP	13,281	4,699	6,625	264	28,180
Loan	13,281	6,986	12,730	487	56,620
CPI	13,281	1.020	0.017	1	1.057

### Empirical Strategy

The econometric model is constructed as follows,


(1) $$\begin{array}{r} Med_{ict}=\alpha +\beta \cdot AQI_{ct}+\theta \cdot X_{ict}+\varepsilon_{ict} \end{array}$$

where Med represents the health care expenses, AQI indicates air quality index, X denotes a series of control variables affecting health care expenses. In Equation (1), subscript i, c, t respectively stand for individuals, cities, and survey years.

Table 1 shows, the average household consumption is RMB 50,241 yuan, which is lower than the household disposable income. The average healthcare expenditure is only 4,957 yuan. The average household size is close to 4. The average child ratio is 12%, the average elderly ratio is 18%, reflecting smaller household size, fewer young people, and the aging population under the birth control policy. The average age of the householder is 52 years old, ranging from 17 to 95 years old. The average years of education of householders are about 8 years, which is equal to junior high school.

**Figure 1:**
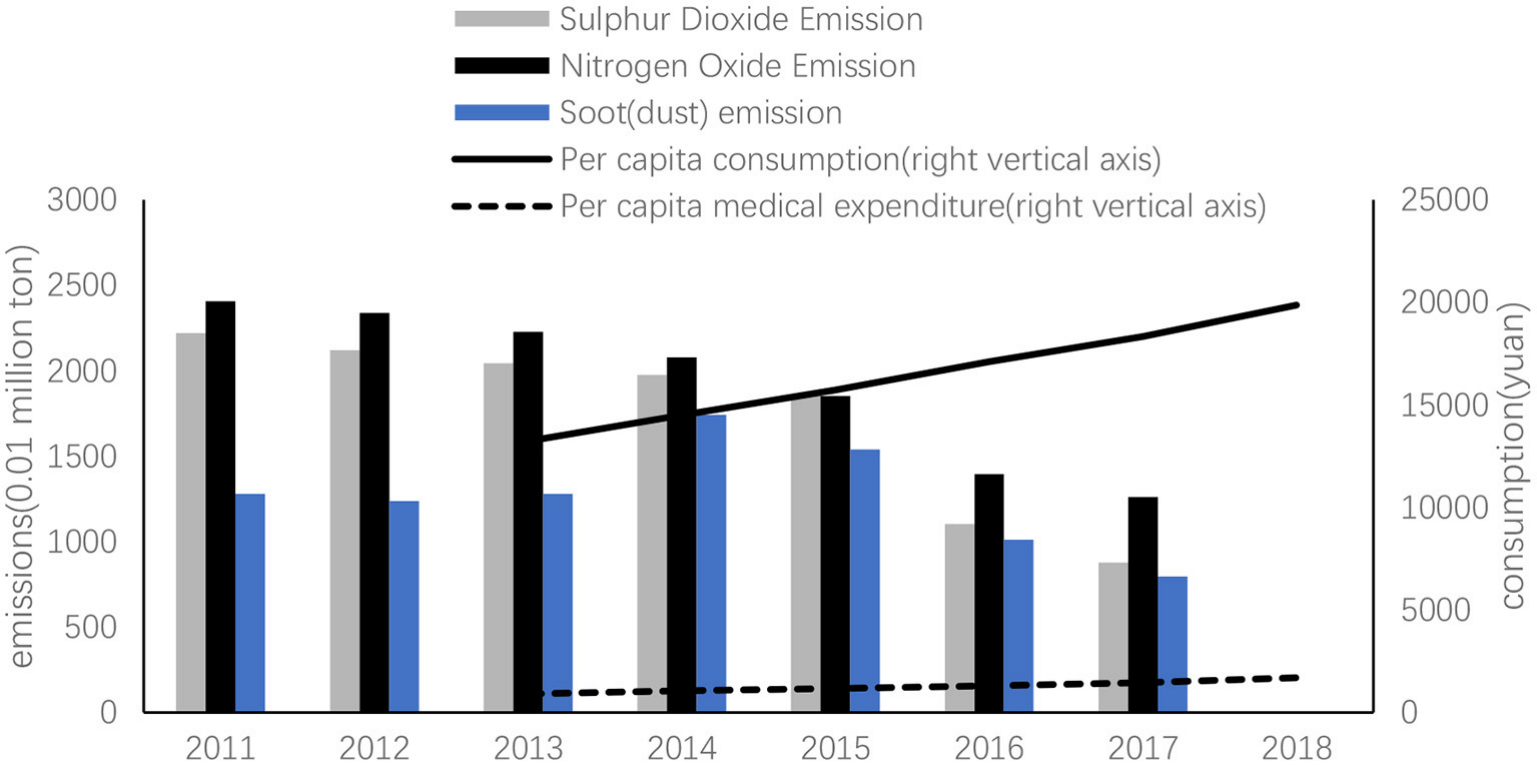
The trend of consumption, medical expenditure, and primary pollutants (2011–2018).

From Figure 1, Per capita consumption rose from RMB 13,220 yuan in 2013 to RMB 19,853 yuan in 2018. Per capita medical expenditure increased from RMB 912 yuan to RMB 1,685 yuan. Since 2013, per capita medical consumption grew much faster than per capita consumption, and the proportion of medical expenditure in total consumption rose steadily. With the continuous improvement of China's economic and social development, the building of ecological civilization also gradually increases, environmental monitoring and regulations are becoming increasingly strict. From 2011 to 2017, SO_2_ Emission, Nitrogen Oxide Emission, and Soot (dust) Emission present a downward trend, as shown in Figure 1.

## Empirical Results

### Baseline Regressions

Table 2 reports the baseline regression results. After controlling individual characteristics, household variables, province and year fixed effect, Column 1 in Table 2 shows that the AQI is positive significantly with total household consumption, which means that the poorer air quality is, the more households will consume. For per one unit adding of AQI, the consumption will increase by about 0.275 yuan. Among total consumption, food consumption and medical expenditure are also positively correlated with AQI. On the one hand, with abnormal weather (such as haze, sandstorms, etc.), malignant pollution accidents, and other incidents occurring frequently, households will prefer high-quality food (such as pollution-free products) and anti-pollution products (such as air purifiers) and seek better living conditions for health reasons, thus increasing their food expenditure. On the other hand, environmental pollution will induce residents to be sick. Households will also spend more to prevent the negative impact of environmental pollution on their health, thus improving the medical expenditure.

**Table 2:** Air pollution and household medical expenses: baseline regressions.

	**(1)**	**(2)**	**(3)**	**(4)**	**(5)**	**(6)**	**(7)**	**(8)**	**(9)**
	**ln** ***pce***	**ln** ***daily***	**ln** ***dress***	**ln** ***eec***	**ln** ***food***	**ln** ***house***	**ln** ***med***	**ln** ***other***	**ln** ***trco***
AQI	0.275^**^	0.200	0.284	0.317	0.434^***^	0.155	1.363^**^	−0.203	0.408
	(0.135)	(0.302)	(0.373)	(0.724)	(0.155)	(0.262)	(0.533)	(0.428)	(0.261)
CPI	−3.694	−8.558^*^	−8.324	−31.199^***^	−1.111	−3.359	−26.413^***^	−35.693^***^	−8.754^**^
	(2.256)	(4.935)	(6.025)	(11.626)	(2.548)	(4.317)	(8.532)	(7.489)	(4.236)
Age	−0.008^**^	−0.024^***^	0.009	0.046^***^	−0.001	0.015^**^	−0.018	−0.042^***^	0.030^***^
	(0.003)	(0.007)	(0.009)	(0.016)	(0.003)	(0.008)	(0.012)	(0.010)	(0.008)
Age^2^/100	0.002	0.009	−0.033^***^	−0.074^***^	−0.004	−0.016^**^	0.034^***^	0.029^***^	−0.049^***^
	(0.003)	(0.007)	(0.009)	(0.015)	(0.004)	(0.007)	(0.012)	(0.010)	(0.008)
Eduy	0.025^***^	0.027^***^	0.034^***^	0.125^***^	0.027^***^	0.021^***^	0.037^***^	0.059^***^	0.042^***^
	(0.002)	(0.004)	(0.004)	(0.008)	(0.002)	(0.003)	(0.006)	(0.005)	(0.003)
Gender	−0.053^***^	−0.120^***^	−0.112^**^	−0.243^***^	−0.006	−0.053^**^	−0.006	−0.075^*^	−0.022
	(0.013)	(0.029)	(0.035)	(0.067)	(0.015)	(0.026)	(0.050)	(0.042)	(0.025)
Marry_dum	0.153^***^	0.146^***^	0.106^*^	−0.241^**^	0.184^***^	0.153^***^	0.535^***^	0.163^***^	0.277^***^
	(0.020)	(0.043)	(0.057)	(0.093)	(0.022)	(0.043)	(0.076)	(0.062)	(0.044)
Health_dum	−0.072^***^	0.009	0.201^***^	0.263^**^	0.065^***^	−0.035	−1.172^***^	0.145^***^	0.077^**^
	(0.016)	(0.036)	(0.049)	(0.081)	(0.019)	(0.031)	(0.053)	(0.052)	(0.034)
Work_dum	−0.114^***^	0.157^***^	−0.031	0.148^*^	−0.216^***^	−0.080^***^	−0.191^***^	0.018	0.073^**^
	(0.015)	(0.035)	(0.043)	(0.079)	(0.017)	(0.031)	(0.061)	(0.050)	(0.031)
Social_insurance	−0.049^*^	−0.029	−0.020	−0.172	−0.066^**^	0.011	0.245^**^	0.110	−0.017
	(0.022)	(0.051)	(0.063)	(0.115)	(0.026)	(0.049)	(0.096)	(0.077)	(0.048)
Ownership	−0.216^***^	−0.290^***^	−0.288^***^	−0.497^***^	−0.187^***^	−0.339^***^	−0.161^**^	−0.382^***^	−0.224^***^
	(0.018)	(0.042)	(0.050)	(0.094)	(0.021)	(0.046)	(0.075)	(0.061)	(0.036)
Urban	0.201^***^	0.061^**^	0.061^*^	0.642^***^	0.334^***^	0.284^***^	−0.132^**^	0.229^***^	0.039
	(0.014)	(0.030)	(0.036)	(0.070)	(0.016)	(0.028)	(0.051)	(0.043)	(0.026)
Risk_prefer	0.072^***^	0.088^**^	0.040	−0.015	0.133^***^	0.068^**^	−0.194^***^	0.168^***^	0.062^**^
	(0.017)	(0.037)	(0.045)	(0.087)	(0.018)	(0.033)	(0.066)	(0.053)	(0.031)
Familysize	0.085^***^	0.066^***^	0.147^***^	0.460^***^	0.062^***^	0.083^***^	0.187^***^	0.008	0.135^***^
	(0.004)	(0.009)	(0.010)	(0.022)	(0.005)	(0.009)	(0.015)	(0.014)	(0.008)
Ln*income*	−0.851^***^	−1.272^***^	−0.389^*^	−1.354^***^	−0.388^***^	−0.348^**^	−0.141	−1.313^***^	−0.425^***^
	(0.070)	(0.150)	(0.192)	(0.298)	(0.079)	(0.144)	(0.245)	(0.199)	(0.143)
Ln*income*^2^	0.055^***^	0.081^***^	0.042^***^	0.092^***^	0.031^***^	0.028^***^	0.016	0.090^***^	0.036^***^
	(0.004)	(0.008)	(0.010)	(0.016)	(0.004)	(0.007)	(0.013)	(0.010)	(0.007)
Ln*total_asset*	0.107^***^	0.218^***^	0.159^***^	0.228^***^	0.083^***^	0.140^***^	0.068^***^	0.200^***^	0.163^***^
	(0.006)	(0.013)	(0.016)	(0.025)	(0.006)	(0.013)	(0.020)	(0.018)	(0.012)
Ln*house_debt*	0.015^***^	0.023^***^	0.003	0.000	0.001	0.039^***^	0.021^***^	0.013^***^	0.009^***^
	(0.002)	(0.003)	(0.003)	(0.007)	(0.002)	(0.003)	(0.005)	(0.004)	(0.002)
Ln*nonhousing_debt*	0.024^***^	0.038^***^	−0.000	0.020^**^	0.001	0.012^***^	0.053^***^	0.030^***^	0.020^***^
	(0.002)	(0.004)	(0.004)	(0.009)	(0.002)	(0.003)	(0.006)	(0.005)	(0.003)
Old_ratio	−0.074^**^	−0.251^***^	−0.783^***^	−0.055	0.004	−0.132^**^	0.703^***^	−0.364^***^	−0.639^***^
	(0.029)	(0.064)	(0.095)	(0.133)	(0.032)	(0.057)	(0.104)	(0.091)	(0.068)
Child_ratio	−0.044	0.137	0.221^**^	4.793^***^	0.186^***^	−0.119	−0.026	0.103	−0.234^***^
	(0.041)	(0.093)	(0.091)	(0.212)	(0.045)	(0.083)	(0.159)	(0.132)	(0.069)
Green	0.005^***^	0.009^***^	0.004	0.014^***^	0.003^***^	0.005^**^	0.004	0.004	0.008^***^
	(0.001)	(0.002)	(0.002)	(0.005)	(0.001)	(0.002)	(0.003)	(0.003)	(0.001)
Loan_gdp	0.061^***^	0.053	−0.055	0.203^***^	0.086^***^	0.095^***^	0.101^*^	−0.144^***^	0.078^***^
	(0.014)	(0.030)	(0.034)	(0.071)	(0.016)	(0.024)	(0.055)	(0.050)	(0.022)
Province	Yes	Yes	Yes	Yes	Yes	Yes	Yes	Yes	Yes
Year	Yes	Yes	Yes	Yes	Yes	Yes	Yes	Yes	Yes
Observations	12,924	13,172	13,148	13,237	13,222	13,156	13,281	13,252	13,104
*R* ^2^	0.497	0.303	0.344	0.321	0.455	0.209	0.098	0.236	0.426

*Standard errors in parentheses*;

* *p < 0.10*,

** *p < 0.05*,

*** *p < 0.01*.

Concerning the control variables, we utilize the income square as a proxy for income uncertainty. As shown in Table 2, the coefficient of household income square is significantly positive with consumption. It can be clear that there is a non-linear relationship between income and consumption. When household income is lower, the precautionary saving theory can explain this. According to the precautionary saving theory, the more income uncertainty an individual is confronted with, the less likely to spend. While household income is larger than the threshold, it turns to be positively correlated with consumption.

Furthermore, we examine the effect of AQI on the consumption structure. According to the classification standards of the National Bureau of Statistics, this paper divides the total consumption into eight categories: food, clothing, daily supplies and services, medical care, transportation and communication, education and cultural services, housing, and other supplies and services. The dependent variables are the proportions of various spending in total consumption expenditure. The results^2^ show that similar to Table 2, AQI is still significantly and positively correlated with food spending and health care expenditure.

We further perform the following robust tests to test the robustness of the conclusions displayed in Table 2. We then give the regression results after replacing the core explanatory variables.^3^ The AQI is calculated mainly based on the six pollutants, PM2.5, PM10, SO_2_, NO_2_, O_3_, and CO. Results show that the explanatory variables we focus on are still significantly positive with household consumption. Excluding the PM2.5 and SO_2_, the rising concentration of PM10 will substantially stimulate medical expenditure.

### Heterogeneities

With the frequent environmental destructions and the occurrences of abnormal weather (such as haze, sandstorms, etc.) and vicious pollution accidents, residents will prefer high-quality food (such as pollution-free products) and anti-polluted devices (such as air purifiers) and seek a better living environment, and finally leading to the increasing of consumer spending. Generally speaking, individuals in cities with higher economic development will consume much more than those in the underdeveloped countryside. What's more, urban and rural residents are exposed unequally to environmental pollutions. Schoolman and Ma (13) find that Chinese villages with a higher proportion of rural migrant workers are more likely to be exposed to severe air and water pollution. Therefore, we divide the sample into urban and rural areas and perform the regressions, respectively. Columns 1, 2 in Table 3 show that residents in urban areas are much more sensitive to air pollution than rural areas.

**Table 3:** Impact of Air pollution on household medical expenses: by area, years of education, and gender.

	**ln** ***med***							
	**Urban**	**Rural**	**eduy ≤ 6**	**6 < eduy ≤ 9**	**9 < eduy ≤ 12**	**Eduy > 12**	**Male**	**Female**
AQI	1.590^**^	1.237^*^	0.996	1.463	0.892	4.412^**^	1.232^*^	1.404^*^
	(0.772)	(0.745)	(0.808)	(0.895)	(1.451)	(2.066)	(0.736)	(0.772)
Control	Yes	Yes	Yes	Yes	Yes	Yes	Yes	Yes
Province	Yes	Yes	Yes	Yes	Yes	Yes	Yes	Yes
Year	Yes	Yes	Yes	Yes	Yes	Yes	Yes	Yes
Observations	6,733	6,548	5,413	4,673	2,106	1,089	6,750	6,531
*R* ^2^	0.108	0.101	0.104	0.106	0.123	0.150	0.105	0.098

*Standard errors in parentheses*;

* *p < 0.10*,

** *p < 0.05. Control variables are the same as that in Table 2*.

With the intensifying air pollutions and the improvement of household income and educations in various regions of China, people are increasingly aware of the fragility of the ecological environment. Then, how will air pollution produce a heterogeneous effect on health care expenditure for individuals with different education levels? Based on the years of education, the sample is divided into four groups: primary school and below (the years of schooling is six or below), junior high school (the years of education is between 6 and 9), senior high school (the years of education is between 9 and 12), college or above (the years of education is beyond 12). Columns 3–6 in Table 3 show the regression results. The results show that only householders with college or above are sensitive to air pollution. This explanation is that more educated residents will care for their body status more, understanding the harmful consequences of air pollution. Therefore, they are inclined to spend more on health care expenditure.

The gender difference may be closely connected with the ability and awareness to cope with air pollution. Neumayer and Plümper (14) utilize the panel data from 141 countries and find that environmental disasters will significantly decrease women's life expectancy than men. For countries where women have higher socioeconomic status, gender inequality is smaller. Columns 7, 8 in Table 3 show the regression results based on the gender of the householder. The coefficients of AQI in the female group (0.548 for lnpce and 1.404 for lnmed) are much more significant than the corresponding coefficients of the male group (0.001, which is not substantial for lnpce and 1.232 for lnmed). The results indicate that women are more sensitive to air pollution.

Income level may affect households' response to air pollution. Hamilton (15) finds that lower-income and minorities are exposed to a higher risk of hazardous waste. With the increasing air pollution, the householder's health condition has deteriorated, resulting in declining working hours and income. However, if households have higher income, they will have more ways to avoid air pollution, including better awareness and more equipment to deal with pollution. Dividing the income into three groups from low to high, Columns 1–3 in Table 4 shows that the medical expenses of higher-income and lower-income households are more sensitive to air pollution. In contrast, middle-income families are less susceptible to air pollution.

**Table 4:** Impact of air pollution on household medical expenses: by income and with response sensibility index.

	**ln** ***med***					
	**Lower income**	**Middle income**	**High income**	**Low sensibility**	**Middle sensibility**	**High sensibility**
AQI	2.203^*^	−0.317	2.502^**^	−0.147	4.144^*^	7.259^***^
	(0.914)	(0.916)	(0.964)	(2.430)	(2.353)	(2.553)
Control	Yes	Yes	Yes	Yes	Yes	Yes
Province	Yes	Yes	Yes	Yes	Yes	Yes
Year	Yes	Yes	Yes	No	No	No
Observations	4,506	4,421	4,354	1,539	1,546	1,558
*R* ^2^	0.105	0.119	0.089	0.109	0.157	0.139

*Standard errors in parentheses*;

* *p < 0.10*,

** *p < 0.05*,

*** *p < 0.01. Control variables are the same as that in Table 2*.

Furthermore, we apply the response sensibility index in 144 cities put forward by Zheng et al. (16). A larger index indicates that the city's Weibo sentiment is more affected by changes in air quality. Dividing the response sensibility index into three groups from low to high, columns 4–6 in Table 4 shows that households in cities with a higher response sensibility index are more sensitive to air pollution. The more individuals pay attention to environmental issues, the more they tend to increase health care expenditure.

### Mechanism

Then how air pollution affects household consumption? One channel is through environmental awareness. Generally, there is a positive correlation between environmental pollution and public environmental awareness. When the environment where residents live is seriously polluted, it will affect their daily life, and it will inevitably evoke the public's awareness of protecting the environment. Inglehart (17) finds that residents support environmental protection in more polluted countries and regions through the World Values Survey data (WVS). Franzen (18) points that countries with relatively more prominent environmental problems, such as Russia, Turkey, and the Czech Republic, are more concerned about environmental conditions than other countries, and residents have higher environmental awareness. Meanwhile, regions with severe environmental pollutions may receive more support to take relevant measures for solving environmental problems (19, 20).

Environmental awareness affects the environmental behaviors of residents (21). From the perspective of consumption choices, compared with traditional products, although green products are more expensive, more and more people are willing to pay a higher premium for environmentally friendly products (22, 23). The stronger consumers' environmental awareness is, the more willing they are to pay higher prices for environmentally friendly products (24). The questionnaire in CFPS asks respondents their opinions about current environmental problems in China, and their answers range from not serious to very serious, with values ranging from 1 to 10. Therefore, this paper divides the sample into three groups by pollution awareness, Not bad group with pollution awareness is <3, Bad group with pollution awareness is larger than 3 and less than 6, Severe group with pollution awareness is larger than 6. Columns 1–3 in Table 5 shows that there is a positive relationship between air pollution and medical expenditure. When individuals realize that environmental pollution is more severe, they will dramatically increase healthcare spending. Therefore, enhancing environmental awareness is expected to be the key to stimulating sustainable consumption behaviors (25).

**Table 5:** Impact of air pollution on household medical expenses: pollution awareness, health status, depression and risk attitude.

	**Lnmed**					**Depress**	**Lnmed**	**Lnmed**	
	**Not bad**	**Bad**	**Severe**	**Health**	**Unhealth**			**Risk_prefer = 1**	**Risk_prefer = 0**
AQI	0.512	0.544	2.244^***^	1.896^***^	0.046	−1.709^***^	2.179^***^	0.491	1.621^***^
	(1.581)	(0.961)	(0.755)	(0.631)	(1.077)	(0.561)	(0.744)	(1.408)	(0.576)
Depress							−0.083^***^		
							(0.016)		
Control	Yes	Yes	Yes	Yes	Yes	Yes	Yes	Yes	Yes
Province	Yes	Yes	Yes	Yes	Yes	Yes	Yes	Yes	Yes
Year	Yes	Yes	Yes	Yes	Yes	Yes	Yes	Yes	Yes
Obs	1,626	4,157	5,949	9,645	2,087	6,634	6,634	2,182	11,099
*R* ^2^	0.131	0.105	0.110	0.079	0.097	0.171	0.109	0.112	0.099

*Standard errors in parentheses*;

*** *p < 0.01. Control variables are the same as that in Table 2*.

Among existing research about the impact of air pollution on health care expenditure, it is generally believed that the risk of diseases will arise when the air quality deteriorates, which will evoke the demand for commercial medical insurance (4). Air pollution is harmful to infants and young children (26, 27) and negatively affects the health of adults (28, 29). Ebenstein et al. (30) find that the annual average concentration of PM10 arises owing to coal heating policies in northern areas of the Huaihe River in China, resulting in the shortened life expectancy of north residents, using the regression discontinuity design. Columns 4, 5 in Table 5 reports the results based on whether the householder is healthy or not. The results show that, compared with householders with poor health conditions, when the householders are in good health, the rising of AQI will produce a more significant positive effect on the medical consumption. Again it proves that poor self-reported health is associated with an increase in air pollution index (31).

Outdoor environments such as weather and pollutions will affect our mood and behaviors. Evans et al. (32) point that those being exposed to severely polluted environments will aggravate their depression, anxiety, and tension. Li et al. (33) study households who live around the Jinchuan mining area in China and find that possible health effects of air pollution reduce their happiness. We use the depression variable to represent an individual emotional status, with values from 1 to 5. CFPS questionnaire asks respondents about their frequency of depression in the last month, answers successively decrease from almost every day, often, half-time, sometimes to never. Columns 6, 7 in Table 5 show that the regressions using depression as an intermediate variable. When depression is included in Column two, the coefficient of AQI on health care expenditure is significantly positive, indicating that air pollution will partly affect health care spending through individuals' emotional status. Persson et al. (34) point that people with higher trait anxiety scores should express more annoyance when asked about their annyance reactions to exposures from specific environmental elements.

On the other hand, air pollution may affect household economic decision-making behaviors through their risk attitude (35). Levy and Yagil (36) analyze data from more than 2,000 trading days on four U.S. stock exchange markets, discover that emotional variations caused by air pollution can lead to risk aversion and reductions of stock returns. Hu et al. (37) analyze stock trading and AQI in cities, finding that the local AQI (characterizing the concentration of pollutants) higher than Beijing negatively affects local stock prices and trading volume. Columns 8, 9 in Table 5 give the results of risk attitudes. The results show that when individuals are more risk-averse, they are more inclined to increase healthcare expenditure to avoid the adverse effects of air pollution on their health.

### Endogeneity Discussion

Identifying the causal relationship between air pollution and health care expenditure and adequately addressing the endogeneity of air pollution are the main challenges in empirical analysis. The endogeneity issues are mainly due to the following reasons. First is omitted variables. There may exist some unobservable omitted variables that both affecting air pollution and health care spending. Even though the temporal and regional effects are controlled, and many control variables are included, this endogeneity cannot be eliminated and bias the estimation results. Second, there are measurement errors concerning air pollution indicators. Existing literature finds that to reduce the pressure of environmental assessment, local governments will not improve the environmental situation but play a data game (38). Air pollution statistics may be suspected of falsification and impair data quality. Meanwhile, if the pollution data are searched around the nearest monitoring point where the individuals lived, using this kind of data source as the air pollution will lead to measurement errors. It can be seen that solving the endogenous problems of air pollution is the prerequisite and basis for accurately estimating the impact of air pollution on health care expenditure.

Therefore, based on Hering and Poncet (39), this paper uses cities' annual air flowing coefficient as instrumental variables for air pollution. This instrument variable depends on the city's current wind speed and the height of the planetary boundary layer, both of which are determined by meteorological and geographical conditions. Table 6 reports the two-stage instrumental variable regression results. Column 1 in Table 6 shows, the air flowing coefficient is statistically significant, and the F statistic is greater than 10, the hypothesis of weak instrumental variables is rejected. Column 2 in Table 6 shows that AQI is still positively correlated with the medical expenses after considering the endogeneity.

**Table 6:** Instrumental variables regression results.

	**First stage**	**Second stage**	**First stage**	**Second stage**	**First stage**	**Second stage**	**First stage**	**Second stage**
	**AQI**	**ln** ***med***	**AQI100_num**	**ln** ***wmed***	**AQI150_num**	**ln** ***med***	**AQI200_num**	**ln** ***med***
AQI		14.253^***^						
		(5.053)						
AQI100_num				−0.224				
				(0.186)				
AQI150_num						0.127^**^		
						(0.051)		
AQI200_num								0.149^***^
								(0.055)
Lnac (IV)	−0.012^***^		0.783		−1.383^***^		−1.175^***^	
	(0.001)		(0.592)		(0.302)		(0.160)	
Control	Yes	Yes	Yes	Yes	Yes	Yes	Yes	Yes
Year	Yes	Yes	Yes	Yes	Yes	Yes	Yes	Yes
Observations	13,281	13,281	13,281	13,281	13,281	13,281	13,281	13,281
*R* ^2^	0.107	0.021	0.192	–	0.1637	–	0.1464	–

*Standard errors in parentheses*;

** *p < 0.05*,

*** *p < 0.01. Control variables are the same as that in Table 2*.

Given China's official definition, AQI with being larger than 100, 150, and 200, represent different pollution levels. Therefore, we further calculate the number of days for cities with AQI greater than 100, 150, and 200. Columns 3–8 in Table 6 show that, except that the days with AQI greater than 100 are not statistically significant, days with AQI greater than 150 and days with AQI greater than 200 both boost health care spending.

There are arrangements to implement differential heating in North and South cities in China based on the “Qinling-Huaihe” geographical boundary. This is equivalent to an intervention experiment in which a group of cities is arranged approximately randomly on either side of the boundary (40). The unique heating in winter for northern cities uses coal as the primary heating energy source, causing the inferior air quality of the northern cities due to pollutant emissions. Based on the panel data, we compare health care expenditure changes in cities near the north and south sides of the Qinling-Huaihe geographic boundary to examine the impact of air pollution. We report the regression results of the cities located in the north and south of the Qinling-Huaihe geographic boundary.^4^ Results show that AQI significantly promotes health care expenditure of residents in northern cities. In contrast, AQI in northern cities still positively affects total consumption expenditures, although it is not statistically significant.

## Conclusions

By matching AQI data at the city level with household data, we discuss the impact of air pollution on household consumption, especially medical and health care spending from a micro perspective. We find that air pollution is significantly positive with household medical and health care expenses. This effect is still significant after controlling for relevant household and individual characteristics and economic characteristics. Under different educational backgrounds, income, hukou, gender, and other conditions, air pollution still significantly affects consumer spending. For those females in the urban areas with higher education backgrounds and higher income, the spending elasticity of air pollution is larger than other corresponding groups. Furthermore, we find that the impact of air pollution on medical and health care spending is particularly significant in cities north of the Qinling Ridge-Huaihe River Line.

Considering that China government has implied a series of environmental regulation policies to alleviate air pollution, our finding also has implications for policymakers. First, considering the impact of environmental regulations on individual consumption behaviors, the government should minimize the crowding-out effect of air pollution on consumption. When implementing environmental policies like levying more environmental pollution taxes and fees on areas with severe environmental pollution, the government should consider corporate responses and develop relevant policies for residents, including consumption subsidies and preferential taxation. Second, the government should pay attention to the different influences of air pollution on individuals with various conditions. The impact of air pollution on the consumption of different individuals and the future welfare effect should be given more attention when formulating environmental regulatory policies.

## Data Availability Statement

Publicly available datasets were analyzed in this study. This data can be found here: http://www.isss.pku.edu.cn/cfps/download; http://www.stats.gov.cn/; http://www.mee.gov.cn/.

## Author Contributions

LZ contributed to the idea, completed the empirical test and analysis, and wrote the manuscript. QZ reviewed the paper and gave some important revision suggestions. JY performed the data analyses and manuscript preparation. All authors contributed to the article and approved the submitted version.

## Funding

This study is supported by the National Office for Philosophy and Social Sciences (CN) (Grant No. 20CJL031) and the Department of Science and Technology of Guangdong Province (Project Nos. 2020A1515010413 and 2019A101002054).

## Conflict of Interest

The authors declare that the research was conducted in the absence of any commercial or financial relationships that could be construed as a potential conflict of interest.

## Publisher's Note

All claims expressed in this article are solely those of the authors and do not necessarily represent those of their affiliated organizations, or those of the publisher, the editors and the reviewers. Any product that may be evaluated in this article, or claim that may be made by its manufacturer, is not guaranteed or endorsed by the publisher.

## Footnotes

^1^As the length limit, variable definitions are given in Appendix Table 1 in Supplementary Material.

^2^As the length limit, regression results are given in in Appendix Table 2 in Supplementary Material.

^3^As the length limit, regression results are given in Appendix Table 3 in Supplementary Material.

^4^As the length limit, regression results are given in Appendix Table 4 in Supplementary Material.
