# Supplementary material for: Air Pollution and Household Medical Expenses: Evidence From China
Source: Front Public Health. 2022 Feb 7;9:798780. doi: 10.3389/fpubh.2021.798780 (PMC8859457; doi:10.3389/fpubh.2021.798780)
Supplement: Supplementary file 1 [file Table_1.DOCX]

Supplementary Material

# Supplementary Tables

# Appendix Table 1 Variable Definitions

| Variable Name | Definition |
| --- | --- |
| Lnpce | The logarithm of total household consumption in last year (Unit: yuan) |
| Lnmed | The logarithm of household health care expenditure in last year (Unit: yuan) |
| Lndaily | The logarithm of household daily necessities and services expenditure in last year (Unit: yuan) |
| Lndress | The logarithm of household clothing consumption in last year (Unit: yuan) |
| Lneec | The logarithm of household education, culture, and entertainment in last year (Unit: yuan) |
| Lnfood | The logarithm of household food expense in last year (Unit: yuan) |
| Lnhousing | The logarithm of household housing expense in last year (Unit: yuan) |
| Lntrco | The logarithm of household transportation and communications expense in last year (Unit: yuan) |
| Lnother | The logarithm of household other goods and services in last year(Unit: yuan) |
| AQI | Air quality index annually |
| PM2.5 | PM25 annually(Unit: mg/m^3^) |
| PM10 | PM10 annually(Unit: mg/m^3^) |
| SO2 | SO2 annually(Unit: mg/m^3^) |
| NO2 | NO2 annually(Unit: mg/m^3^) |
| CO | CO annually(Unit: mg/m^3^) |
| O3 | O3 annually(Unit: mg/m^3^) |
| AQI100_num | the days with AQI>=100 |
| AQI150_num | the days with AQI>=150 |
| AQI200_num | the days with AQI>=200 |
| Age | the age of householder |
| Eduy | the years of householder educated |
| Work_dum | Whether the householder is employed or not, 1=employed; 0=unemployed |
| Health_dum | Whether the householder is healthy or not, 1=healthy; 0=unhealthy |
| Social_insurance | Whether the householder has social insurance or not, 1=secured; 0=unsecured |
| Urban | Whether the household is urban or not, 1=urban; 0=rural |
| Marry_dum | Householder’s marry status, 1=married; 0=unmarried |
| Gender | Householder’s gender, 1=male; 0=female |
| Ownership | Whether the household own a house,1=house owner; 0=not having houses |
| Risk_prefer | Householder’s risk attitude, 1=risk preference; 0=risk aversion |
| Child_ratio | The ratio of children number with age less than 15 to household members |
| Old_ratio | The ratio of elder number with age more than 65 to household members |
| Familysize | The number of household member |
| Lnincome | The logarithm of total household income in last year (Unit: yuan) |
| Lntotal_asset | The logarithm of total household asset in current year (Unit: yuan) |
| Lnhouse_debt | The logarithm of total household outstanding mortgage in current year (Unit: yuan) |
| Lnnonhouse_debt | The logarithm of total household outstanding non-mortgage in current year (Unit: yuan) |
| Green | Percentage of greenery coverage in city level(Unit: %) |
| GDP | Gross domestic product in city level in current year (Unit: 0.1 billion yuan) |
| Loan | Loans of financial institutions credit in city level and current year (Unit: 0.1 billion yuan) |
| CPI | Consumer price index, which is set to 1 in 2014 as the base year |

**Appendix Table 2** Air pollution and eight categories of consumption structure

|  | (1) | (2) | (3) | (4) | (5) | (6) | (7) | (8) |
| --- | --- | --- | --- | --- | --- | --- | --- | --- |
|  | ln*daily* | ln*dress* | ln*eec* | ln*food* | ln*house* | ln*med* | ln*other* | ln*trco* |
| AQI | -0.141 | 0.012 | -0.069 | 0.236^*^ | -0.136 | 0.876^*^ | -0.491 | 0.203 |
|  | (0.269) | (0.360) | (0.699) | (0.127) | (0.236) | (0.521) | (0.425) | (0.246) |
| Lnpce | 1.078^***^ | 0.734^***^ | 1.613^***^ | 0.655^***^ | 0.914^***^ | 1.079^***^ | 0.732^***^ | 0.672^***^ |
|  | (0.021) | (0.027) | (0.045) | (0.011) | (0.020) | (0.034) | (0.030) | (0.018) |
| CPI | -3.155 | -4.896 | -27.542^**^ | 1.877 | -0.185 | -21.043^**^ | -33.904^***^ | -7.313^*^ |
|  | (4.362) | (5.846) | (11.258) | (2.087) | (3.822) | (8.336) | (7.389) | (3.978) |
| Age | -0.014^**^ | 0.017^**^ | 0.062^***^ | 0.005^*^ | 0.023^***^ | -0.010 | -0.034^***^ | 0.037^***^ |
|  | (0.006) | (0.009) | (0.015) | (0.003) | (0.007) | (0.011) | (0.010) | (0.007) |
| Age^2/100 | 0.006 | -0.037^***^ | -0.082^***^ | -0.007^**^ | -0.018^***^ | 0.031^***^ | 0.025^***^ | -0.051^***^ |
|  | (0.006) | (0.009) | (0.015) | (0.003) | (0.007) | (0.011) | (0.010) | (0.008) |
| Eduy | -0.001 | 0.016^***^ | 0.084^***^ | 0.010^***^ | -0.003 | 0.009 | 0.041^***^ | 0.024^***^ |
|  | (0.003) | (0.004) | (0.008) | (0.002) | (0.003) | (0.006) | (0.005) | (0.003) |
| Gender | -0.064^**^ | -0.078^**^ | -0.167^***^ | 0.028^**^ | -0.006 | 0.033 | -0.045 | 0.005 |
|  | (0.026) | (0.034) | (0.064) | (0.012) | (0.023) | (0.048) | (0.041) | (0.024) |
| Marry_dum | -0.024 | -0.015 | -0.483^***^ | 0.080^***^ | 0.009 | 0.368^***^ | 0.050 | 0.170^***^ |
|  | (0.038) | (0.056) | (0.090) | (0.018) | (0.038) | (0.074) | (0.061) | (0.042) |
| Health_dum | 0.085^**^ | 0.246^***^ | 0.342^***^ | 0.111^***^ | 0.026 | -1.087^***^ | 0.195^***^ | 0.120^***^ |
|  | (0.033) | (0.047) | (0.079) | (0.016) | (0.028) | (0.050) | (0.052) | (0.032) |
| Work_dum | 0.280^***^ | 0.050 | 0.331^***^ | -0.142^***^ | 0.012 | -0.097^*^ | 0.098^*^ | 0.151^***^ |
|  | (0.031) | (0.042) | (0.077) | (0.014) | (0.028) | (0.059) | (0.050) | (0.030) |
| Social_insurance | 0.035 | 0.009 | -0.080 | -0.033 | 0.060 | 0.294^***^ | 0.120 | 0.033 |
|  | (0.045) | (0.060) | (0.110) | (0.021) | (0.045) | (0.095) | (0.075) | (0.046) |
| Ownership | -0.053 | -0.123^**^ | -0.118 | -0.038^**^ | -0.146^***^ | 0.072 | -0.215^***^ | -0.074^**^ |
|  | (0.037) | (0.049) | (0.090) | (0.017) | (0.042) | (0.072) | (0.060) | (0.034) |
| Urban | -0.153^***^ | -0.083^**^ | 0.330^***^ | 0.201^***^ | 0.093^***^ | -0.363^***^ | 0.090^**^ | -0.096^***^ |
|  | (0.027) | (0.035) | (0.068) | (0.013) | (0.025) | (0.051) | (0.043) | (0.024) |
| Risk_prefer | 0.007 | -0.017 | -0.122 | 0.084^***^ | 0.002 | -0.273^***^ | 0.115^**^ | 0.010 |
|  | (0.033) | (0.044) | (0.083) | (0.015) | (0.029) | (0.064) | (0.052) | (0.029) |
| Familysize | -0.026^***^ | 0.082^***^ | 0.321^***^ | 0.004 | 0.005 | 0.096^***^ | -0.056^***^ | 0.079^***^ |
|  | (0.008) | (0.010) | (0.021) | (0.004) | (0.008) | (0.015) | (0.014) | (0.007) |
| Ln*income* | -0.369^***^ | 0.255 | -0.012 | 0.166^**^ | 0.391^***^ | 0.777^***^ | -0.719^***^ | 0.145 |
|  | (0.134) | (0.185) | (0.289) | (0.065) | (0.134) | (0.237) | (0.196) | (0.135) |
| Ln*income^2* | 0.023^***^ | 0.001 | 0.004 | -0.004 | -0.020^***^ | -0.043^***^ | 0.051^***^ | -0.001 |
|  | (0.007) | (0.009) | (0.015) | (0.003) | (0.007) | (0.012) | (0.010) | (0.007) |
| Ln*total_asset* | 0.103^***^ | 0.081^***^ | 0.048^**^ | 0.010^**^ | 0.043^***^ | -0.053^***^ | 0.121^***^ | 0.090^***^ |
|  | (0.011) | (0.015) | (0.023) | (0.005) | (0.012) | (0.019) | (0.017) | (0.011) |
| Ln*house_debt* | 0.007^***^ | -0.008^**^ | -0.024^***^ | -0.008^***^ | 0.025^***^ | 0.005 | 0.002 | -0.001 |
|  | (0.003) | (0.003) | (0.007) | (0.001) | (0.002) | (0.005) | (0.004) | (0.002) |
| Ln*nonhousing_debt* | 0.012^***^ | -0.017^***^ | -0.020^**^ | -0.015^***^ | -0.010^***^ | 0.027^***^ | 0.014^***^ | 0.004 |
|  | (0.003) | (0.004) | (0.008) | (0.002) | (0.003) | (0.006) | (0.005) | (0.002) |
| Old_ratio | -0.169^***^ | -0.737^***^ | 0.069 | 0.061^**^ | -0.068 | 0.802^***^ | -0.308^***^ | -0.570^***^ |
|  | (0.060) | (0.093) | (0.129) | (0.026) | (0.051) | (0.098) | (0.091) | (0.066) |
| Child_ratio | 0.199^**^ | 0.257^***^ | 4.868^***^ | 0.231^***^ | -0.075 | 0.021 | 0.103 | -0.206^***^ |
|  | (0.082) | (0.088) | (0.206) | (0.037) | (0.076) | (0.154) | (0.129) | (0.063) |
| Green | 0.004^**^ | 0.002 | 0.006 | -0.000 | 0.000 | -0.000 | 0.002 | 0.004^***^ |
|  | (0.002) | (0.002) | (0.004) | (0.001) | (0.002) | (0.003) | (0.003) | (0.001) |
| Loan_gdp | -0.012 | -0.094^***^ | 0.096 | 0.043^***^ | 0.033 | 0.041 | -0.202^***^ | 0.035^*^ |
|  | (0.026) | (0.033) | (0.068) | (0.013) | (0.022) | (0.053) | (0.049) | (0.021) |
| Province | Yes | Yes | Yes | Yes | Yes | Yes | Yes | Yes |
| Year | Yes | Yes | Yes | Yes | Yes | Yes | Yes | Yes |
| Observations | 12924 | 12860 | 12924 | 12905 | 12924 | 12924 | 12923 | 12831 |
| R^2^ | 0.464 | 0.396 | 0.387 | 0.637 | 0.381 | 0.169 | 0.277 | 0.499 |

Note: Standard errors in parentheses；^*^ *p* < 0.10, ^**^ *p* < 0.05, ^***^ *p* < 0.01.

**Appendix Table 3** Impact of air pollution on household medical expenses: with different core explanatory variables

|  | ln*pce* | | | ln*med* | | |
| --- | --- | --- | --- | --- | --- | --- |
| PM2.5 | 0.253^*^ |  |  | 0.579 |  |  |
|  | (0.130) |  |  | (0.517) |  |  |
| PM10 |  | 0.277^**^ |  |  | 1.358^***^ |  |
|  |  | (0.114) |  |  | (0.441) |  |
| SO_2_ |  |  | 0.314^***^ |  |  | -0.343 |
|  |  |  | (0.120) |  |  | (0.442) |
| Control | Yes | Yes | Yes | Yes | Yes | Yes |
| Province | Yes | Yes | Yes | Yes | Yes | Yes |
| Year | Yes | Yes | Yes | Yes | Yes | Yes |
| Observations | 12924 | 12924 | 12924 | 13281 | 13281 | 13281 |
| R^2^ | 0.497 | 0.497 | 0.497 | 0.097 | 0.098 | 0.097 |

Note: Standard errors in parentheses；^*^ *p* < 0.10, ^**^ *p* < 0.05, ^***^ *p* < 0.01. Control variables are the same as that in Table 2.

**Appendix Table 4** Impact of Air pollution on household medical expenses: by Qinling Ridge-Huaihe River

|  | ln*pce* | | ln*med* | |
| --- | --- | --- | --- | --- |
|  | North cities | South cities | North cities | South cities |
| AQI | 0.223 | 0.420 | 1.622^*^ | 0.089 |
|  | (0.192) | (0.292) | (0.841) | (1.060) |
| Control | Yes | Yes | Yes | Yes |
| Province | Yes | Yes | Yes | Yes |
| Year | Yes | Yes | Yes | Yes |
| Observations | 5493 | 3943 | 5613 | 4143 |
| R^2^ | 0.481 | 0.495 | 0.106 | 0.098 |

Note: Standard errors in parentheses；^*^ *p* < 0.10, ^**^ *p* < 0.05, ^***^ *p* < 0.01. Control variables are the same as that in Table 2.
